# Effects of Dietary Sugar Reduction on Biomarkers of Cardiometabolic Health in Latino Youth: Secondary Analyses from a Randomized Controlled Trial

**DOI:** 10.3390/nu15153338

**Published:** 2023-07-27

**Authors:** Kelsey A. Schmidt, Pari Mokhtari, Elizabeth A. Holzhausen, Tanya L. Alderete, Hooman Allayee, Krishna S. Nayak, Frank R. Sinatra, Trevor A. Pickering, Wendy Mack, Rohit Kohli, Michael I. Goran

**Affiliations:** 1 Department of Pediatrics, The Saban Research Institute, Children’s Hospital Los Angeles, 4650 Sunset Boulevard, Mailstop #61, Los Angeles, CA 90027, USA; kelschmidt@chla.usc.edu (K.A.S.); pmokhtari@chla.usc.edu (P.M.); 2 Department of Integrative Physiology, University of Colorado Boulder, Boulder, CO 80309, USA; elizabeth.holzhausen@colorado.edu (E.A.H.); tanya.alderete@colorado.edu (T.L.A.); 3 Department of Population and Public Health Sciences, University of Southern California, Los Angeles, CA 90033, USA; hallayee@usc.edu (H.A.); tpickeri@usc.edu (T.A.P.); wmack@usc.edu (W.M.); 4 Ming Hsieh Department of Electrical and Computer Engineering, University of Southern California, Los Angeles, CA 90089, USA; knayak@usc.edu; 5 Alfred E. Mann Department of Biomedical Engineering, University of Southern California, Los Angeles, CA 90089, USA; 6 Department of Pediatrics, University of Southern California, Los Angeles, CA 90033, USA; sinatra@med.usc.edu; 7 Division of Gastroenterology and Hepatology, Department of Pediatrics, Children’s Hospital Los Angeles, Los Angeles, CA 90027, USA; rokohli@chla.usc.edu

**Keywords:** Latino, glucose tolerance, obesity, sugar, metabolic disease, adolescents

## Abstract

Pediatric obesity and cardiometabolic disease disproportionately impact minority communities. Sugar reduction is a promising prevention strategy with consistent cross-sectional associations of increased sugar consumption with unfavorable biomarkers of cardiometabolic disease. Few trials have tested the efficacy of pediatric sugar reduction interventions. Therefore, in a parallel-design trial, we randomized Latino youth with obesity (BMI ≥ 95th percentile) [*n* = 105; 14.8 years] to control (standard diet advice) or sugar reduction (clinical intervention with a goal of ≤10% of calories from free sugar) for 12-weeks. Outcomes included changes in glucose tolerance and its determinants as assessed by a 2-h frequently sample oral glucose tolerance test, fasting serum lipid profile (total cholesterol, HDL, LDL, triglycerides, cholesterol:HDL), and inflammatory markers (CRP, IL-6, TNF-α). Free sugar intake decreased in the intervention group compared to the control group [11.5% to 7.3% vs. 13.9% to 10.7% (% Energy), respectively, *p* = 0.02], but there were no effects on any outcome of interest (*p*_all_ > 0.07). However, an exploratory analysis revealed that sugar reduction, independent of randomization, was associated with an improved Oral-disposition index (*p* < 0.001), triglycerides (*p* = 0.049), and TNF-α (*p* = 0.02). Dietary sugar reduction may have the potential to reduce chronic disease risks through improvements in beta-cell function, serum triglycerides, and inflammatory markers in Latino adolescents with obesity.

## 1. Introduction

Pediatric obesity continues to rise, with rates disproportionately impacting Latino children [1]. This is concerning because childhood obesity is associated with metabolic complications including dyslipidemia, high blood pressure, and impaired glucose tolerance [2]. As a result, Latino children are at an even greater risk for developing chronic diseases such as cardiovascular disease (CVD) and type 2 diabetes (T2D) [3]. Therefore, it is imperative to identify key intervention strategies to reduce the prevalence of chronic disease risk factors, especially in Latino youth.

Reducing the consumption of added sugars, particularly in the form of sugar sweetened beverages (SSBs), has been identified as one modifiable dietary factor that has the potential to improve the metabolic profile in children and adolescents. Cross-sectional studies in adolescents consistently find associations between sugar consumption and markers of glucose tolerance and insulin sensitivity [4,5,6,7,8,9,10], with the most consistent evidence for positive associations between sugar intake and the homeostatic model of insulin sensitivity (HOMA-IR). Further, sugar reduction has been implicated as a strategy to reduce CVD risk in at-risk pediatric populations, and the American Heart Association has issued a statement indicating that there is strong evidence to support the association between added sugar intake and increased CVD risk in children [10]. Sugar intake has also been associated with blood pressure [6,8,11,12] and the fasting serum lipid profile in pediatric populations [7,8,12,13]. It is also important to note that some studies identified that outcomes differ by participant’s sex and race or ethnicity [5,8,9,12]. Overall, there is consistent and strong evidence of associations between sugar intake and markers of cardiometabolic health.

While few randomized controlled trials (RCTs) have tested the efficacy of sugar reduction as a strategy to improve blood pressure, serum lipid profile, or glucose tolerance and its determinants in children and adolescents, the trials show promise. In a school-based intervention in Brazilian 9–12-year-olds, children attending schools randomized to receive education that discouraged soft drink intake had significant decreases in fasting glucose and total cholesterol compared to children at control schools [14]. A pilot study conducted by our research group randomized 16 overweight female Latina adolescents to receive a dietary intervention through one-on-one weekly counseling or through group counseling sessions. In both groups, the dietary intervention focused on sugar reduction (<10% of total calories) and increased fiber intake (14 g/1000 calories). The change in added sugar intake was associated with change in area under the curve (AUC) for insulin through a 3-h oral glucose tolerance test (OGTT) [15]. However, limitations of this study include the small sample size, that the study was only conducted in females, and that the study lacked an adequate comparator group. A follow-up study randomized 54 overweight male and female Latino adolescents to either a control, nutrition (weekly nutrition classes), or nutrition plus strength training group for 16-weeks. Although this study found no overall significant intervention, participants who specifically decreased their added sugar intake (regardless of intervention group) had improvements in AUC glucose and AUC insulin [16,17]. The same intervention was also conducted in overweight African American adolescents. These results showed significant group by ethnicity interactions, indicating that African American participants had significant increases in 2-h glucose, AUC insulin, and AUC glucose as compared to decreases seen in Latino populations; as well as an increase in acute insulin response to glucose [18]. Therefore, while there is preliminary evidence indicating that sugar reduction is an efficacious strategy to improve cardiometabolic profiles in children and adolescents, further trials are needed to confirm these results.

To further explore whether a dietary intervention focused on sugar reduction impacts the cardiometabolic profile, we randomized Latino adolescents with obesity to either a control group receiving standard dietary advice or an intervention group that received dietary counseling focused on sugar reduction and home-delivery of bottled water with a goal of reducing added sugar intake to 10% or less of their total calories. Outcomes of interest included measures of glucose tolerance and its determinants, the fasting serum lipid profile, and markers of inflammation. We hypothesized that participants in the intervention group would have greater improvements in cardiometabolic health. 

## 2. Materials and Methods

### 2.1. Recruitment and Enrollment

Detailed recruitment and enrollment procedures, including key inclusion and exclusion criteria, were previously described [19]. We enrolled Latino adolescents (11–18 years of age) with obesity (BMI ≥ 95th percentile for age and sex) from the greater Los Angeles area into this trial. All eligible and enrolled participants signed youth assents and their parents signed informed consents prior to the study’s initiation. The USC and CHLA institutional review boards approved this study. This trial was registered on clinicaltrials.gov on 28 October 2016 (NCT02948647) before enrolling the first study participant.

### 2.2. Study Design

The study design, including randomization, study diets, and pre-specified study outcomes, were previously published [19]. Briefly, this 12-week parallel-design randomized controlled trial randomized subjects in a one-to-one ratio to either a sugar reduction intervention group (intervention group) or a control group receiving handouts with general diet advice (control group) for 12 weeks. Pre- and post-intervention assessments were conducted at the Diabetes and Obesity Research Institute’s (DORI) clinical facility at USC. The results for the primary and selected secondary outcomes of this study, including liver fat (primary outcome), liver fibrosis, and anthropometrics, were previously published [19]. Results from the pre-specified secondary endpoints including glucose tolerance and its determinants, blood pressure, fasting lipid profile, adipokines and inflammatory markers are presented here.

### 2.3. Study Diets

Specifics of the intervention and control diets were previously presented [19]. In summary, participants randomized to the control group received a packet of handouts with general dietary advice based on the 2015 Dietary Guidelines for Americans [20] and the United States Department of Agriculture MyPlate [21]. Participants randomized to the intervention group met regularly with a bilingual registered dietitian nutritionist (RDN) to discuss principles of healthy eating, with a focus on reducing free sugar intake [intake of added sugar and sugar from sugar-sweetened beverages (SSBs)]. Intervention participants were also asked to monitor their free sugar intake and were provided with a free sugar intake maximum of 10% of their daily calories, as per the recommendations by the 2015–2020 Dietary Guidelines for Americans [20]. Further, intervention participants received a weekly delivery of bottled water in an effort to displace SSBs. All participants were asked to continue their usual physical activities and were not provided with any specific calorie targets.

### 2.4. Glucose Tolerance and Determinants of Glucose Tolerance

At both clinic visits, participants completed a fasting blood draw and 2-h frequently samples oral glucose tolerance test (FS-OGTT) after a 10-h overnight fast. Secondary outcome measures included glucose tolerance as assessed by fasting glucose and the area under the curve (AUC) glucose; systemic insulin sensitivity as assessed by fasting insulin, the Homeostatic Model Assessment of Insulin Resistance (HOMA-IR), AUC insulin, and the Matsuda-De-Fronzo insulin sensitivity index (ISI) [22]; Pancreatic *β*-cell function as assessed by the insulinogenic index [23] and the oral disposition index (oral DI) [24], all based on the 2-h FS-OGTT. Glucose was assayed in duplicate using the glucose oxidase method and a Yellow Springs instrument 2300 analyzer (YSI Inc., Yellow Springs, OH, USA). Insulin and C-peptide were assayed in duplicate using the Millipore ELISA kits (EMD Millipore Inc., Burlington, MA, USA). Glucose excursion was further evaluated by changes in glycated hemoglobin (HbA1c). HbA1c was measured in fasting whole blood by the Norris lab at USC using non-porous ion exchange high performance liquid chromatography on a Tosoh G8 analyzer.

### 2.5. Fasting Serum Lipid Profile & Biomarkers

The fasting serum lipid profile, adipokines, and inflammatory markers were measured in fasting plasma by the DORI Metabolic Core Laboratory. The fasting serum lipid profile included triglycerides, total cholesterol, low-density lipoprotein (LDL) cholesterol, high-density lipoprotein (HDL) cholesterol, and the total cholesterol:HDL cholesterol ratio. Serum lipids were assayed using FujiFilm kits utilizing the microplate method (FUJIFILM Wako Diagnostics, Mountain View, CA, USA). The intra- and inter-assay coefficient of variation (CV) for the HDL-Cholesterol E assay is 8.23% and 3.71%, respectively; the intra- and inter-CV for the Cholesterol E assay is 8.80% and 7.75%, respectively; and the intra- and inter-assay CV for the L-Type Triglyceride M assay is 8.23% and 3.71%, respectively. Adipokines, including Monocyte Chemoattractant Protein-1 (MCP-1) and leptin, as well as markers of low-grade chronic systemic inflammation as assessed by concentrations of interleukin-6 (IL-6), and TNF-Alpha were measured in fasting plasma using the Millipore Magpix Metabolic Panel kit at the DORI Core Laboratory (intra-assay and inter-assay CV of 4.53% and 6.96%, respectively). High-sensitivity C-reactive protein (CRP) was assayed separately using the Millipore ELISA kits, with an intra- and inter-assay CV of 4.60% and 6.00%, respectively.

### 2.6. Anthropometrics

Body weight and height were measured by a registered nurse or phlebotomist using standardized procedures [25,26].

### 2.7. Dietary Intake and Physical Activity

The methods used to assess dietary intake and physical activity were described previously [19]. Briefly, dietary intake was assessed by 24-h dietary recalls and data was collected and compiled using the Nutrition Data System for Research (versions 2016–2019, University of Minnesota). Physical activity was measured by metabolic equivalents calculated from data captured by a 3-day physical activity recall that was based on the validated Previous Day Physical Activity Recall [27].

### 2.8. Statistical Analysis

We aimed to randomize 120 participants into the trial. This sample size was calculated based on the primary aim of the trial, change in liver fat [19].

Statistical analyses were performed using the R statistical package version 4.0.3 [28] and RStudio version 1.4.1106 (RStudio, Inc., Boston, MA, USA) [29], with the 2-sided level of significance set to *p* < 0.05 for all analyses. All variables were assessed for normality and logarithmic transformations were performed on all variables that showed considerable right-skewedness. Per CONSORT guidelines, baseline characteristics were visualized by a randomization arm for the size of any chance imbalances [30]. The secondary outcomes reported here were calculated as the change (post minus pre) in the outcomes of interest. Secondary outcomes included measures of glucose tolerance (AUC glucose, fasting glucose, 2-h glucose, HbA1c), insulin sensitivity (HOMA-IR, fasting insulin, Matsuda ISI, AUC insulin), beta-cell function (insulinogenic index and Oral DI), fasting serum lipids (total cholesterol, triglycerides, HDL cholesterol, LDL cholesterol, cholesterol:HDL), blood pressure (systolic and diastolic), and inflammatory markers (TNF-α, IL-6, MCP-1, CRP). For all outcomes, values greater than or equal to three standard deviations from the mean were removed from the analysis to reduce the potential of spurious results caused by outliers. The main effects of the intervention were evaluated using general multivariable linear models, with change in outcome as the dependent variable, adjusting for the outcome variable at the baseline (raw model). We subsequently ran an adjusted model that included covariates for sex, change in body mass index (BMI), and change in physical activity (adjusted model). To determine whether the treatment effect differed by participant sex (male vs. female), we added an interaction term of sex-by-treatment arm. The main analysis included those individuals with complete data for the outcome of interest. In an additional analysis, we also used generalized estimating equations (GEE) to adjust for clustering of sibling pairs who were enrolled into the study and had complete data at both the baseline and post-intervention assessments. In the analysis of dietary data, four subjects were removed because they may have received dietary advice from the study team before completing their baseline dietary assessment.

In exploratory analyses of the pooled data, we assessed the impact of reducing sugar intake, irrespective of randomization, on glucose tolerance and its determinants, the fasting serum lipid profile, blood pressure, and markers of inflammation. To assess the effect of reducing total sugar intake on our outcomes of interest, we split our data set into those with a reduction in total sugar over the intervention period (TS; TS_Post (% of energy)_ − TS_Pre (% of energy)_ < 0) and those with no change or an increase in total sugar intake (TS_Post (% of energy)_ − TS_Pre (% of energy)_ ≥ 0) during the intervention period, regardless of study diet assignment. This variable replaced the diet intervention variable in the general linear models described above.

## 3. Results

### 3.1. Description of Participants and Adverse Events

**Figure 1:**
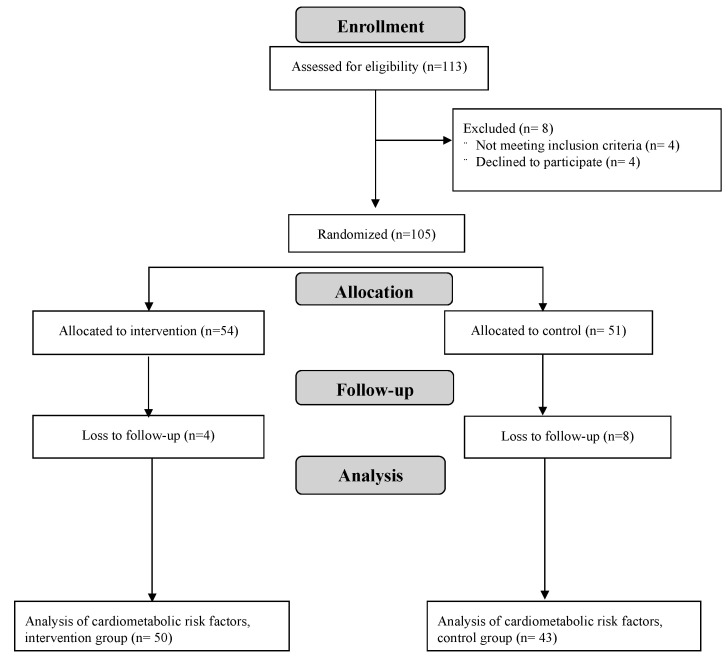
Flow diagram of participant recruitment, enrollment, intervention allocation, follow-up, and analysis.

One hundred and thirteen potential participants were assessed for eligibility with 105 participants enrolled and randomized into the trial. Participants were randomly assigned to either the dietitian-led sugar reduction intervention group (*n* = 54) or the control group (*n* = 51) receiving handouts with general diet advice (Figure 1). Twelve participants dropped out before the post-intervention assessment and were excluded from the analyses of outcome variables of interest. Table 1 presents the baseline characteristics for our study population, stratified by intervention group. No major imbalances between intervention groups were identified. Baseline characteristics for all participants enrolled and randomized into the trial were previously reported [19]. No adverse events were reported, related or un-related to the intervention, during the conduct of the trial.

### 3.2. Intervention Adherence and Dietary Intakes

Intervention adherence in our study population was previously described [19]. To summarize, while there was no significant difference in free sugar intake between the control and intervention groups at baseline (*p* = 0.35, Table S1), at the post-intervention assessment, those randomized to the intervention group consumed significantly less energy from free sugars than the control group (*p* = 0.003). Further, a higher percentage of individuals randomized to the intervention group as compared to the control group reduced their free sugar intake and met the intervention target of a free sugar intake ≤ 10% of total calories. Additionally, in alignment with the design of our study, participants generally remained weight-stable with no notable change in their total energy intake (*p* = 0.67) or weight (*p* = 0.48) over the intervention period.

We previously reported dietary intake data for the 88 participants who were randomized with complete diet data [19]. Briefly, the intervention group had a greater reduction in energy adjusted total sugar intake (*p* < 0.01), added sugar intake (*p* = 0.02), and free sugar intake (*p* < 0.01) compared to the control group (Table S1). There were no statistically significant differences in the intakes of total energy or nutrient adjusted carbohydrates, fiber, fat, saturated fatty acids, monounsaturated fatty acids, polyunsaturated fatty acids, or protein between the intervention and control groups (*p*_all_ > 0.10).

### 3.3. Glucose Tolerance and Determinants of Glucose Tolerance

There were no differential effects of our intervention as compared to the control group on changes in glucose tolerance as measured by fasting glucose (adjusted *p* = 0.21), 2-h glucose (adjusted *p* = 0.25), HbA1c (adjusted *p* = 0.16), or AUC glucose (adjusted *p* = 0.58) (Table 2). There was also no differential effect of our intervention on measures of insulin sensitivity including fasting insulin (adjusted *p* = 0.36), 2-h insulin (adjusted *p* = 0.44), HOMA-IR (adjusted *p* = 0.23), Matsuda ISI (adjusted *p*= 0.20), or AUC insulin (adjusted *p* = 0.38) (Table 2). No differential effects on beta-cell function were indicated, including no differential changes in the insulinogenic index (adjusted *p* = 0.34) or oral DI (adjusted *p* = 0.26) (Table 2). There was no evidence of an intervention by sex interaction for changes in fasting glucose, 2-h glucose, AUC glucose, 2-h insulin, Matsuda ISI, AUC insulin, insulinogenic index, or oral DI (Table 2, sex interaction *p* > 0.05 for all endpoints). There was a significant intervention by sex interaction for changes in HbA1c (adjusted *p =* 0.02), with HbA1c increasing in the control group as compared to the intervention group among males and HbA1c decreasing in the control group as compared to the intervention group among females (Figure S1). There was also an intervention by sex interaction for HOMA-IR (adjusted *p* = 0.03) (Table 2). However, the sex interaction for HOMA-IR was attenuated and no longer statistically significant in sensitivity analyses that removed values more than two standard deviations from the mean. Our GEE analysis of glucose tolerance and determinants of glucose tolerance yielded consistent results (Table 2).

### 3.4. Blood Pressure and Fasting Serum Lipids

We observed no differential effects of our intervention on diastolic blood pressure (adjusted *p* = 0.46), systolic blood pressure (adjusted *p* = 0.49), or fasting serum lipids including total cholesterol (adjusted *p* = 0.40), triglycerides (adjusted *p* = 0.64), LDL cholesterol (adjusted *p* = 0.30), HDL cholesterol (adjusted *p* = 0.37), or the cholesterol:HDL ratio (adjusted *p* = 0.65) in either the raw or adjusted models (*p* > 0.10 for all endpoints) (Table 3). However, there was an intervention by sex interaction for both diastolic and systolic blood pressure (*p* < 0.05 and *p* < 0.01, respectively). Specifically, there were greater improvements in systolic blood pressure among control males compared to intervention males, whereas there were increases in systolic blood pressure among control females as compared to intervention females. Similar outcomes were observed for diastolic blood pressure, though to a lesser degree. Consistent results were obtained in our GEE analysis of all blood pressure and fasting serum lipid endpoints (*p* > 0.10 for all endpoints).

### 3.5. Inflammatory Markers & Adipokines

Change in adipokines did not differ between the intervention and control groups including no statistically significant differential changes in MCP-1 (adjusted *p* = 0.38) or leptin (adjusted *p* = 0.70) (Table 4). Additionally, the change in the inflammatory markers including CRP and TNF-α did not differ significantly between the intervention and control groups (adjusted *p* = 0.38 and 0.52, respectively). However, there was a trend for a statistically significant difference in the change in IL-6, with a greater increase in the control group as compared to the intervention group (adjusted *p* = 0.07). There was no significant intervention by sex interactions identified (*p*_all_ > 0.05). The GEE analysis of changes in adipokines and markers of systemic inflammation were consistent for all endpoints (*p* > 0.10), except the trend for a differential change in IL-6 was notably attenuated (*p* = 0.24).

### 3.6. Changes in Cardiometabolic Health Outcomes as a Function of Change in Total Sugar Intake Regardless of Intervention Group Assignment

In exploratory analyses of the pooled data, we examined glucose tolerance and its determinants, blood pressure, fasting serum lipids, inflammation, and adipokines in all participants who successfully reduced sugar intake (*n* = 63) relative to participants without sugar reduction (*n* = 28) regardless of intervention assignment. On average, those in the sugar reduction group reduced their total sugar intake by 7.7% of total energy for an average total sugar intake of 13.6 ± 5.2% of total energy after 12 weeks of study participation. On average, those participants who did not reduce their sugar intake increased their sugar intake by 5.9% of total energy for an average total sugar intake of 20.8 ± 6.7% total energy after 12 weeks of study participation.

**Figure 2:**
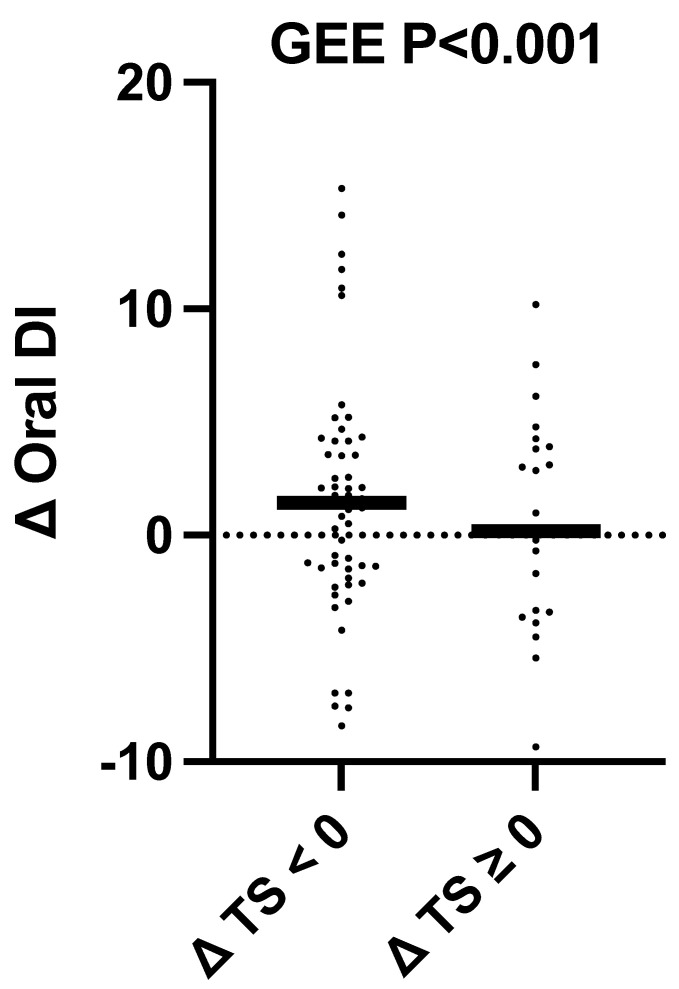
Oral-DI increases in participants with sugar reduction. Changes (post-intervention minus the value at baseline) in the oral disposition index (Oral-DI) for participants with total sugar reduction as a percent of energy (∆TS<0) and those without (∆TS≥0). Each participant’s change variable is represented by a solid dot. The medians are represented by horizontal bars. The *p*-value for the generalized estimating equation adjusted for sex, change in body mass index, change in physical activity, and sibling clusters is displayed at the top of the plot. GEE: generalized estimating equation.

There was no significant difference in the change of fasting glucose, 1-h glucose, HbA1c, AUC glucose, fasting insulin, 2-h insulin, HOMA-IR, Matsuda ISI, AUC insulin, or the Insulinogenic Index between participants who reduced total sugar intake versus those who did not (Table S1) and no sex interactions (*p*_all_ > 0.10). While there was no difference between sugar responder groups for change in oral DI in the ANCOVA analysis, there was a significant 23% increase/improvement in the oral DI in individuals with sugar reduction, as opposed to only an 8.8% increase in those without, in the generalized estimating equation analysis that accounted for sibling pairs (*p* < 0.001, Figure 2). However, it should be noted that this result was no longer significant after running a sensitivity analysis removing individuals more than two standard deviations from the mean.

**Figure 3:**
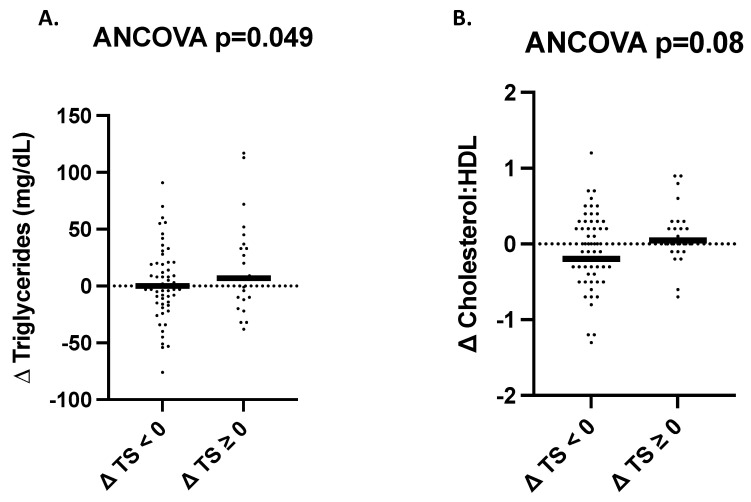
Triglycerides and the Cholesterol to HDL ratio is lower in participants with sugar reduction. Changes (post-intervention minus the value at baseline) in Triglycerides (**A**) and the Cholesterol to HDL ratio (**B**) for participants with total sugar reduction as a percent of energy (∆TS<0) and those without (∆TS≥0). Each participant’s change variable is represented by a solid dot. The medians are represented by horizontal bars. The *p*-value for the analysis of covariance adjusted for sex, change in body mass index, and change in physical activity is displayed at the top of each plot. ANCOVA: analysis of co-variance.

For fasting serum lipids, there was no difference in the change in total cholesterol, LDL cholesterol, or HDL cholesterol between participants with or without sugar reduction. However, there was a significant increase in total triglycerides among those without sugar reduction as compared to those with sugar reduction (*p* = 0.049) and a trend for an increase in the cholesterol to HDL ratio among those without sugar reduction as compared to a decrease in those who reduced their sugar intake (*p* = 0.08, Figure 3, Table S2). There were no significant differences in systolic or diastolic blood pressure between participants who reduced total sugar intake versus those who did not (*p*_all_ > 0.01). There was no intervention by sex interactions for any measure of fasting serum lipids.

There were no statistically significant differences in changes in inflammatory markers or adipokines with the exception of a greater increase in TNF-α among the group without sugar reduction as compared to those who reduced their sugar intake (*p* = 0.02) (Figure S2). There was no intervention by sex interactions for any of the measured inflammatory markers or adipokines (Table S4).

## 4. Discussion

A dietitian-led intervention with the goal of reducing free sugar intake to ≤10% of total calories did not differentially improve glucose tolerance and its determinants, the fasting serum lipid profile, blood pressure, or inflammatory markers when compared to receiving general dietary advice in Latino adolescents with obesity. While the intervention group had a greater reduction in sugar intake than the control group, both groups reduced their sugar intake, with only a 3% difference in total calories from total sugar between groups. Therefore, it may be that the difference in total sugar intake between groups was too small to significantly differentially alter the cardiometabolic outcomes of interest.

Given that we saw sugar reductions in both our intervention and control groups, we also conducted an exploratory analysis that more specifically looked at the impact of sugar reduction on our outcomes of interest by comparing participants with and without total sugar reduction, independent of randomization. Our exploratory results indicate that reducing sugar intake as an intervention strategy may still hold promise for improving cardiometabolic health for at-risk Latino youth. Specifically, we found that participants who successfully reduced their sugar intake saw improvements in their oral-DI as compared to participants who did not reduce their sugar intake, indicating that they had improvements in their beta-cell function. Specifically, those with sugar reduction improved their oral-DI by 23% as compared to only a 9% improvement in those without sugar reduction. Oral-DI is a sensitive measure of the beta-cell function that measures beta-cell output while adjusting for insulin sensitivity. Oral-DI is a significant biomarker of disease risk and has been indicated to be more important in predicting the future development of type 2 diabetes as compared to fasting and 2-h glucose levels [31]. Our result is consistent with a previous study conducted by our group in overweight 8–13-year-old Latino adolescents, which found that sugar intake explained approximately 12% of the variance observed in oral-DI as measured by an OGTT [9], with increased sugar intake associated with decreased beta-cell function. Now, our current study adds to our understanding by demonstrating that reducing total sugar intake is an effective strategy to improve beta-cell function in at-risk Latino youth. This is important because beta-cell deterioration is hypothesized to be a factor that contributes to the pathogenesis of type 2 diabetes [32]. Therefore, reducing sugar intake may reduce the risk for type 2 diabetes in overweight Latino youth through preservation of the beta-cell function. This is particularly meaningful since Latino children are at an increased risk for type 2 diabetes compared to their white peers [3,33].

In addition to our findings relevant for the risk of type 2 diabetes, our exploratory analysis revealed that participants without sugar reduction had some differences in fasting serum lipids and inflammatory markers as compared to those with sugar reduction. Specifically, we report that those without sugar reduction had a 6.5% increase in triglycerides. This outcome aligns with previous pediatric cohort studies which found that increased sugar intake is positively associated with triglycerides [7,12,34]. Additionally, we report that those without sugar reduction had increased levels of the inflammatory cytokine TNF-α.  While few studies have investigated the association between sugar consumption and markers of inflammation in pediatric populations [35], preliminary evidence is beginning to link sugar intake with inflammatory markers, such as TNF-α in childhood [36]. Therefore, our results are in alignment with hypothesized outcomes based on previous cohort studies. Serum lipids and markers of systemic inflammation, such as TNF-α, are known risk factors for chronic diseases, including cardiovascular disease, one of the leading causes of death in the United States [37]. Therefore, controlling these risk factors through the reduction of sugar intake has important implications as it may serve as a strategy to reduce the risk for chronic disease among Latino adolescents with obesity.

There is one key factor which likely explains the difference in outcomes reported between our primary and exploratory results. As mentioned above, the difference in total sugar intake between the intervention and control groups after 12-weeks was significant but was relatively small at 3% of total calories, and we observed participants with sugar reduction in both groups. In contrast, in the exploratory analysis, the difference in total sugar intake between those who reduced their total sugar intake compared to those who did not was approximately 7% of total calories. Therefore, we conclude that the benefits of a real-world dietary intervention focused on sugar reduction, where participants met with a registered dietitian monthly and receive deliveries of bottled water, does not confer additional improvements in cardiometabolic risk factors compared to participants receiving general diet advice. However, our exploratory results confirm our hypothesis that sugar reduction in at-risk Latino adolescents results in improvements in biomarkers of cardiometabolic risk; specifically, beta-cell function, fasting serum lipids, and the inflammatory marker TNF-*α*. Together, this indicates that new sugar-reduction intervention strategies need to be explored to determine which approach is most efficacious at resulting in meaningful decreases in sugar intake, especially in vulnerable populations such as Latino adolescents.

While we did report some findings from our exploratory analysis, many of the hypothesized changes in cardiometabolic risk factors for glucose tolerance and its determinants, serum lipids, blood pressure, and inflammatory markers were not statistically significant with non-clinically meaningful effect sizes. There are a few potential explanations for these null findings. One is that while there was a 7% difference in sugar intake as a percentage of total calories in our exploratory analysis, this difference may have still been too small to result in hypothesized shifts in these biomarkers. Additionally, while our population was overweight, they may have been too metabolically healthy at baseline to observe statistically significant improvements in some of our outcomes of interest. Further, our study was originally powered based on the study’s primary aim of liver fat, so we may have been underpowered to detect significant changes in some of our secondary and exploratory outcomes of interest. Finally, the study was not blinded, so it may be that participants in the intervention group inaccurately reported their sugar intake to be less than their actual intake, which would have biased results toward the null.

Our study has some important strengths which increase our confidence in our results. First, it assessed glucose tolerance through dynamic testing. Second, we had good participant compliance, with 81.6% of intervention participants reducing their free sugar intake and 71.4% meeting the goal of a free sugar intake maximum of 10% of total calories. In addition, we controlled for changes in fat mass statistically, which provided us with greater certainty that any observed outcomes were due to dietary changes and not changes in body weight or composition. Some limitations of this study include that approximately 35% of participants included in this analysis already met the intervention free sugar intake target of ≤10% of total calories prior to study enrollment. While this decreased our ability to ascertain whether reducing sugar intake through our intervention effects our outcomes of interest, our study still provides key insight into the impact of sugar reduction on our outcomes of interest through our exploratory analysis. Also, given our 12-week study period, we are unable to capture the impact of long-term sustained sugar reduction beyond 3-months. While our study had generally good compliance, following and sustaining a low sugar diet is challenging especially in pediatric populations [38]. Therefore, it remains unclear whether the dietary sugar reduction observed in our study could reasonably be sustained over a period longer than 12-weeks. Further, our study outcomes include biomarkers of cardiometabolic disease and therefore cannot directly ascertain whether the outcomes from this study translate to hypothesized reductions in disease risk. Another limitation is the generalizability of our results to populations other than Latino adolescents with obesity.

In conclusion, our results help fill a critical gap in the literature by further exploring the effects of sugar reduction interventions to improve biomarkers of cardiometabolic disease risk in pediatric populations. Our outcomes indicate that dietary sugar reduction interventions have the potential to reduce the risk of chronic disease development through improvements in beta-cell function, fasting serum triglycerides, and inflammatory markers such as TNF-α in Latino adolescents with obesity, but only under conditions where intervention targets are met. However, it remains unclear which intervention strategies are most effective at producing meaningful and sustainable reductions in sugar intake in pediatric populations. Future studies are warranted with a focus on identifying sustainable intervention strategies that can be implemented in a real-world clinical setting. Additionally, studies are needed in a diverse range of pediatric participant populations with a variety of age ranges, ethnic backgrounds, and metabolic profiles to determine whether the results reported here are reproduced.

## Figures and Tables

**Table 1:** Baseline characteristics of study participants by study arm for individuals who were randomized and completed clinic visit 2 (*n* = 93).

Variable	Intervention (*n* = 50)	Control (*n* = 43)
Age (years)	15.0 (13.0, 16.0)	15.0 (13.0, 17.0)
Male sex (%)	23 (46%)	21 (49%)
Tanner stage ≥ 4 (%) ^1^	35 (70%)	24 (57%)
Body weight (kg)	89.3 ± 18.8	92.7 ± 21.4
BMI (kg/m^2^) ^2^	32.1 (29.3, 37.1)	34.1 (30.3, 37.6)
Fasting glucose (mg/dL)	84.6 ± 7.7	86.3 ± 7.6
Fasting insulin (μU/mL)	25.6 (14.1, 41.3)	25.2 (14.2, 38.7)
2-h glucose (mg/dL)	115.4 ± 20.7	114.6 ± 20.5
2-h insulin (μU/mL)	287.5 (173.6, 519.4)	206.0 (132.6, 386.0)
Glycated hemoglobin (%)	5.5 ± 0.4	5.5 ± 0.3
HOMA-IR	5.0 (3.2, 8.7)	5.4 (3.0, 9.0)
Matsuda ISI	1.2 (0.7, 1.6)	1.2 (0.8, 1.9)
Insulinogenic index	5.7 (4.1, 8.4)	6.2 (4.5, 9.3)
Oral DI	6.8 (4.3, 9.0)	8.7 (4.5, 11.6)
AUC glucose (mg/dL × min)	14,796 ± 2126	14,462 ± 1959
AUC insulin (μU/mL × min)	23,959 (15,675, 34,554)	19,986 (12,631, 283,559)
Cholesterol (mg/dL) ^3^	143 (132, 155)	142 (125, 158)
Triglycerides (mg/dL) ^4^	101 (76, 134)	97 (82, 134)
HDL-Cholesterol (mg/dL) ^5^	40.0 (35.0, 44.0)	39.0 (35.0, 44.0)
LDL-Cholesterol (mg/dL) ^6^	79.5 (67.3, 93.8)	77.0 (65.5, 93.0)
Cholesterol:HDL cholesterol ratio ^3^	3.7 ± 0.9	3.6 ± 1.0
Systolic blood pressure (mmHg)	115 ± 11	116 ± 12
Diastolic blood pressure (mmHg) ^1^	69 ± 7	69 ± 7
C-reactive protein (mg/L) ^4^	1.9 (0.9, 4.6)	1.9 (0.7, 6.2)
Interleukin-6 (pg/mL) ^5^	8.5 (4.6, 44.6)	6.1 (2.3, 19.9)
TNF- α ^7^	2.8 ± 0.9	3.1 ± 1.1
Leptin ^7^	6034 (3460, 10,191)	8657 (4714, 10,658)
MCP-1 ^8^	101 ± 31	107 ± 31
Total energy intake (kcal) ^1^	1451 (1224, 1778)	1488 (1227, 1727)
Total sugar intake (%E) ^1^	18.5 ± 6.6	20.2 ± 6.1
Added sugar intake (%E) ^1^	11.1 ± 6.1	12.6 ± 5.7
Free sugar intake (%E) ^1^	11.7 ± 6.1	13.6 ± 6.7
Free sugar intake > 10% ^1^	31 (62%)	29 (69%)
Physical activity (Met-h/week)	59.1 (55.3, 65.9)	56.8 (53.4, 72.3)

Values are means ± standard deviations, or medians (25th, 75th percentiles) for non-normally distributed variables, or percentages for categorical variables. ^1^ Sample Size: Intervention (*n* = 50), Control (*n* = 42). ^2^ Sample Size: Intervention (*n* = 49), Control (*n* = 42). ^3^ Sample Size: Intervention (*n* = 47), Control(*n* = 43). ^4^ Sample Size: Intervention (*n* = 47), Control (*n* = 40). ^5^ Sample Size: Intervention (*n* = 38), Control (*n* = 37). ^6^ Sample Size: Intervention (*n* = 46), Control (*n* = 43). ^7^ Samples Size: Intervention (*n* = 49), Control (*n* = 42). ^8^ Sample Size: Intervention (*n* = 48), Control (*n* = 42). Abbreviations: AUC: area under the curve, BMI: body mass index, HDL: high density lipoprotein, HOMA-IR: Homeostatic Model Assessment for Insulin Resistance, LDL: low-density lipoprotein, MCP: monocyte chemoattractant protein, TNF: tumor necrosis factor.

**Table 2:** The effect of the intervention on glucose tolerance and its determinants in the primary analysis.

	Intervention		Control		ANCOVA			GEE
	Baseline	Change	Baseline	Change	Raw	Adjusted	Sex Interaction	
Fasting Glucose (mg/dL) ^1^	84.6 ± 7.7	0.3 (−3.0, 3.3)	86.3 ± 7.6	0.5 (−6.2, 5.5)	0.43	0.21	0.91	0.19
2-h glucose (mg/dL) ^2^	115.0 ± 20.7	0.4 ± 19.3	115 ± 20.8	3.9 ± 23.7	0.42	0.25	0.38	0.25
HbA1c (%) ^3^	5.4 ± 0.4	0.0 (−0.1, 0.1)	5.5 ± 0.3	0.0 (−0.1, 0.2)	0.46	0.16	0.02	0.15
AUC glucose (mg/dL × min) ^4^	14,893 ± 2026	−251 ± 1660	14,551 ± 1963	108 ± 1785	0.51	0.58	0.35	0.56
Fasting Insulin (μU/mL) ^5^	23.3 (13.7, 36.9)	−2.1 (−8.7, 4.8)	23.4 (14.1, 37.0)	−0.3 (−11.7, 27.4)	0.39	0.36	0.18	0.25
2-h insulin (μU/mL) ^6^	277.4 (173.5, 514.3)	−63.8 (−150.8, 46.4)	190.5 (132.1, 346.9)	47.9 (−98.7, 124.0)	0.38	0.44	0.92	0.22
HOMA-IR ^7^	5.1 (3.1, 8.8)	−0.4 (−2.2, 0.8)	5.3 (3.1, 8.5)	−0.2 (−2.4, 2.8)	0.28	0.23	0.03	0.27
Matsuda ISI ^8^	1.1 (0.7, 1.6)	0.2 ± 0.8	1.2 (0.7, 1.8)	0.0 ± 0.7	0.11	0.20	0.90	0.14
AUC Insulin (μU/mL × min) ^9^	23,959 (16,285, 34,554)	−2417 (−10,819, 4864)	19,406 (12,351, 25,221)	−131 (−5017, 5458)	0.31	0.38	0.54	0.24
Insulinogenic Index ^10^	5.6 (4.1, 8.3)	0.3 (−1.4, 2.4)	6.2 (4.5, 9.4)	0.1 (−1.3, 1.2)	0.34	0.34	0.28	0.38
Oral DI ^11^	6.4 (4.1, 8.8)	1.8 ± 5.0	7.6 (4.2, 11.2)	0.1 ± 5.1	0.18	0.26	0.53	0.35

Values are means ± standard deviations or medians (25th, 75th percentiles) for non-normally distributed variables. Raw: *p*-values for the ANCOVA analysis adjusted for baseline value of the outcome of interest. Adjusted: *p*-values for the raw model adjusted for sex, change in body mass index, and change in physical activity. GEE: *p*-values for the generalized estimating equation analysis of adjusted model further adjusting for sibling clusters. ^1^ Sample size: Intervention (*n* = 48), Control (*n* = 43), sibling cluster (*n* = 10). ^2^ Sample size: Intervention (*n* = 48), Control (*n* = 41), sibling cluster (*n* = 10). ^3^ Sample size: Intervention (*n* = 47), Control (*n* = 43), sibling cluster (*n* = 10). ^4^ Sample size: Intervention (*n* = 46), Control (*n* = 40), sibling cluster (*n* = 9). ^5^ Sample size: Intervention (*n* = 44), Control (*n* = 39), sibling cluster (*n* = 8). ^6^ Sample Size: Intervention (*n* = 48), Control (*n* = 39), sibling cluster (*n* = 9). ^7^ Sample Size: Intervention (*n* = 48), Control (*n* = 40), sibling cluster (*n* = 9). ^8^ Sample Size: Intervention (*n* = 48), Control (*n* = 38), sibling cluster (*n* = 8). ^9^ Sample size: Intervention (*n* = 45), Control (*n* = 37), sibling cluster (*n* = 8). ^10^ Sample Size: Intervention (*n* = 47), Control (*n* = 41), sibling cluster (*n* = 9). ^11^ Sample size: Intervention (*n* = 45), Control (*n* = 39), sibling cluster (*n* = 8). Abbreviations: ANCOVA: analysis of covariance, AUC: area under the curve, GEE: generalized estimating equation, HbA1c: glycated hemoglobin, HOMA-IR: homeostatic model assessment of insulin resistance, ISI: insulin sensitivity index, oral DI: oral disposition index.

**Table 3:** The effect of the intervention on serum lipids and blood pressure in the modified intent-to-treat analysis.

	Intervention (*n* = 46)		Control (*n* = 43)		ANCOVA			GEE *
	Baseline	Change	Baseline	Change	Raw	Adjusted	Sex Interaction	
Cholesterol (mg/dL)	143.5 (134.2, 155.2)	0.3 ± 13.1	142.0 (125.0, 157.5)	−1.8 ± 14.2	0.59	0.40	0.81	0.50
Triglycerides (mg/dL) ^1^	101.0 (76.0, 134.0)	5.0 (−10.0, 21.0)	96.5 (82.2, 134.2)	0.0 (−15.5, 25.5)	0.77	0.64	0.44	0.63
LDL cholesterol (mg/dL)	80.7 ± 21.5	−0.9 ± 10.8	80.2 ± 23.2	−2.9 ± 12.4	0.39	0.30	0.65	0.36
HDL cholesterol (mg/dL) ^2^	40.0 (35.0, 44.0)	0.3 ± 4.4	39.0 (35.0, 44.0)	−0.6 ± 4.5	0.42	0.37	0.43	0.43
Cholesterol:HDL ^3^	3.7 ± 0.9	0.0 ± 0.4	3.6 ± 1.0	0.0 ± 0.5	0.78	0.65	0.37	0.66
Systolic BP (mmHg) ^4^	115.5 ± 10.7	−1.6 ± 8.8	115.7 ± 11.9	−2.9 ± 9.5	0.54	0.49	<0.01	0.46
Diastolic BP (mmHg) ^5^	68.7 ± 6.4	−0.5 (−3.5, 3.0)	68.9 ± 7.1	−0.5 (−5.0, 4.9)	0.42	0.46	<0.05	0.44

Values are means ± standard deviations or medians (25th, 75th percentiles) for non-normally distributed variables. Raw: *p*-values for the ANCOVA analysis adjusted for baseline value of the outcome of interest. Adjusted: *p*-values for the raw model adjusted for sex, change in body mass index, and change in physical activity. * GEE: *p*-values for the generalized estimating equation analysis of adjusted model further adjusting for sibling clusters (*n* = 10). ^1^ Sample Size: Intervention (*n* = 45), Control (*n* = 42), sibling cluster (*n* = 10). ^2^ Sample Size: Intervention (*n* = 46), Control (*n* = 41), sibling cluster (*n* = 10). ^3^ Sample Size: Intervention (*n* = 47), Control (*n* = 43), sibling cluster (*n* = 10). ^4^ Sample size: Intervention (*n* = 50), Control (*n* = 43), sibling cluster (*n* = 10). ^5^ Sample size: Intervention (*n* = 49), Control (*n* = 42), sibling cluster (*n* = 10). Abbreviations: ANCOVA: analysis of covariance, BP: blood pressure, GEE: generalized estimating equation, HDL: high-density lipoprotein, LDL: low-density lipoprotein.

**Table 4:** The effect of the intervention on adipokines and inflammatory markers in the primary analysis.

	Intervention		Control		ANCOVA			GEE
	Baseline	Change	Baseline	Change	Raw	Adjusted	Sex Interaction	Adjusted
CRP ^1^	1.8 (0.7, 4.5)	−0.1 (−1.0, 1.1)	1.5 (0.7, 5.7)	−0.1 (−0.8, 0.7)	0.37	0.38	0.44	0.37
IL-6 ^2^	9.1 (5.6, 47.2)	0.0 (−3.0, 5.5)	6.6 (2.9, 21.1)	1.0 (−2.3, 3.9)	0.15	0.07	0.40	0.24
TNF- α ^3^	2.8 ± 0.9	0.2 (−0.2, 0.6)	3.1 ± 1.1	0.0 (−0.3, 0.5)	0.25	0.52	0.07	0.37
MCP-1 ^4^	99 ± 27	2.6 ± 18.9	107 ± 31	3.7 ± 26.2	0.60	0.38	0.64	0.66
Leptin ^5^	6034 (3460, 10,191)	−76 ± 2419	8657 (4714, 10,658)	−283 ± 2779	0.96	0.70	0.81	0.61

Values are means ± standard deviations or medians (25th, 75th percentiles) for non-normally distributed variables. Raw: *p*-values for the ANCOVA analysis adjusted for baseline value of the outcome of interest. Adjusted: *p*-values for the raw model adjusted for sex, change in body mass index, and change in physical activity. GEE: *p*-values for the generalized estimating equation analysis of adjusted model further adjusting for sibling clusters. ^1^ Sample size: intervention (*n* = 45), Control (*n* = 39), sibling clusters (*n* = 8). ^2^ Sample size: intervention (*n* = 36), Control (*n* = 34), sibling clusters (*n* = 6). ^3^ Sample size: intervention (*n* = 49), Control (*n* = 40), sibling clusters (*n* = 9). ^4^ Sample size: intervention (*n* = 48), Control (*n* = 43), sibling clusters (*n* = 9). ^5^ Sample size: intervention (*n* = 48), Control (*n* = 42), sibling clusters (*n* = 9). Abbreviations: ANCOVA: analysis of covariance, BMI: body mass index, CRP: *C*-reactive protein, GEE: generalized estimating equations, IL-6: interleukin-6, MCP-1: monocyte chemoattractant protein-1.

## Data Availability

An anonymized dataset including all data described in the manuscript, code book, and analytic code will be made available upon request to the principal investigator (MIG).

## Supplementary Materials

The following supporting information can be downloaded at: https://www.mdpi.com/article/10.3390/nu15153338/s1, Figure S1: Changes in HbA1c differ by participant sex; Figure S2: Changes (post-intervention minus the value at baseline) in TNF-α for participants with total sugar reduction as a percent of energy (∆ TS<0) and those without (∆ TS≥0). Each participant’s change variable is represented by a solid dot. The medians are represented by horizontal bars. The p-value for the analysis of covariance adjusted for the change in body mass index and change in physical activity is displayed at the top of the plot. Table S1: Dietary intakes at baseline and the change in intakes during the intervention period, based on unannounced 24-h dietary recalls, for all participants with complete diet data (*n* = 88); Table S2: The effect of total sugar intake on glucose tolerance and its determinants in the primary analysis. Table S3: The effect of total sugar intake on serum lipids and blood pressure in the primary analysis. Table S4: The effect of total sugar intake on adipokines and inflammatory markers in the primary analysis.

## Abbreviations

Analysis of covariance (ANCOVA), area under the curve (AUC), body mass index (BMI), Children’s Hospital Los Angeles (CHLA), C-reactive protein (CRP), coefficient of variation (CV), cardiovascular disease (CVD), Diabetes and Obesity Research Institute (DORI), fat mass (FM), frequently sampled-oral glucose tolerance test (FS-OGTT), glycated hemoglobin (HbA1c), high-density lipoprotein (HDL), Homeostatic Model Assessment of Insulin Resistance (HOMA-IR), interleukin-6 (IL-6), intent-to-treat (ITT), Matsuda-De-Fronzo insulin sensitivity index (ISI), low-density lipoprotein (LDL), Magnetic Resonance Elastography (MRE), Magnetic Resonance Imaging (MRI), non-alcoholic fatty liver disease (NAFLD), oral disposition index (oral DI), randomized controlled trials (RCT), registered dietitian nutritionist (RDN), sugar sweetened beverages (SSBs), type 2 diabetes (T2D), University of Southern California (USC).
