# Supplementary material for: Effects of Dietary Sugar Reduction on Biomarkers of Cardiometabolic Health in Latino Youth: Secondary Analyses from a Randomized Controlled Trial
Source: Nutrients. 2023 Jul 27;15(15):3338. doi: 10.3390/nu15153338 (PMC10420969; doi:10.3390/nu15153338)
Supplement: Supplementary file 1 [file nutrients-15-03338-s001.zip › nutrients-2487102-supplementary.pdf]

Effects of dietary sugar reduction on biomarkers of cardiometabolic health in Latino youth: secondary analyses from a randomized controlled trial

Schmidt et al. Online Supporting Material

Table S1. Dietary intakes at baseline and the change in intakes during the intervention period, based on unannounced 24-hour dietary recalls, for all participants with complete diet data (n=88)

	**Intervention (n=49)**		**Control (n=39)**		**ANCOVA**			**GEE**
	Baseline	Change	Baseline	Change	Raw	Adjusted	Sex interaction	
Energy intake, kcal/d	1470 (1224, 1791)	-72 ± 502	1509 (1227, 1727)	-75 ± 447	0.79	0.80	0.70	0.68
Carbohydrates, %E	49.8 (44.5, 55.0)	-2.9 ± 8.7	48.0 (45.7, 53.7)	-0.7 ± 11.9	0.25	0.30	0.82	0.32
Total sugar, %E	18.6 ± 6.6	-4.8 ± 7.5	20.0 ± 6.2	-2.1 ± 9.1	<0.01	<0.01	0.84	<0.01
Added sugars, %E	11.1 ± 6.62	-3.7 ± 7.1	13.1 ± 5.5	-2.6 ± 7.5	0.01	0.02	0.74	<0.01
Free sugars, %E	11.7 ± 6.1	-4.8 ± 6.5	13.7 ± 6.5	-3.1 ± 8.3	<0.01	<0.01	0.66	<0.001
Fiber, g/day	13.2 (10.1, 18.1)	-0.6 (-4.0, 3.3)	12.3 (8.2, 15.9)	0.1 (-5.4, 4.0)	0.23	0.21	0.93	0.23
Fiber, %E	3.5 (2.6, 4.2)	-0.1 (-0.9, 1.0)	3.3 (2.5, 4.1)	0.2 (-0.6, 1.0)	0.53	0.54	0.88	0.44
Fat, %E	33.9 ± 7.0	1.4 ± 7.2	32.2 ± 5.3	1.1 ± 9.5	0.14	0.16	0.90	0.17
SFA, %E	11.7 (8.9, 13.3)	0.4 ± 4.0	10.1 (8.9, 12.8)	0.8 ± 4.2	0.78	0.75	0.74	0.76
MUFA, %E	12.1 (10.2, 14.3)	0.7 ± 3.4	10.6 (8.6, 13.1)	0.4 ± 4.6	0.18	0.22	0.76	0.24
PUFA, %E	8.2 (7.2, 10.1)	0.0 ± 3.1	7.1 (5.9, 10.1)	0.2 ± 3.5	0.26	0.28	0.25	0.25
Protein, %E	16.8 ± 4.6	1.5 ± 4.9	17.8 ± 4.6	-0.3 ± 4.8	0.13	0.16	0.19	0.14

Values are means ± standard deviations or medians (25th, 75th percentiles) for non-normally distributed variables

Raw: change adjusted for baseline value

Adjusted: raw model adjusted for sex, change in body mass index, change in physical activity

Abbreviations: ANCOVA: Analysis of covariance, GEE: generalized estimating equation, MUFA: monounsaturated fatty acids, PUFA: polyunsaturated fatty acids, SFA: saturated fatty acids, %E: percent of total energy intake

GEE: generalized estimating equation analysis of adjusted model further adjusting for sibling clusters

Table S2. The effect of total sugar intake on glucose tolerance and its determinants in the primary analysis.

	**Δ total sugar (%E) <0**		**Δ total sugar (%E) ≥ 0**		**ANCOVA**			**GEE**
	Baseline	Change	Baseline	Change	Raw	Adjusted	Sex Interaction	Adjusted
Fasting Glucose (mg/dL)[1]	85.2 ± 7.5	0.2 (-5.8, 4.5)	85.3 ± 8.4	0.9 (-1.9, 3.9)	0.73	0.58	0.64	0.65
2-hour glucose (mg/dL)[2]	115 ± 21	1 ± 22	116 ± 18	3 ± 21	0.65	0.70	0.19	0.69
HbA1c (%)[3]	5.5 ± 0.3	0.0 (-0.1, 0.1)	5.4 ± 0.4	0.0 (-0.1, 0.1)	0.61	0.69	0.87	0.67
AUC glucose (mg/dL x min)[4]	14,719 ± 2,177	-167 ± 1,828	14,708 ± 1,651	33 ± 1,555	0.60	0.56	0.37	0.56
Fasting Insulin (μU/mL)[5]	26.1 (15.9, 36.1)	-0.9 (-11.9, 6.5)	17.4 (13.6, 38.0)	-1.7 (-9.2, 2.7)	0.97	0.93	0.25	0.82
2-hour insulin (μU/mL)[6]	239.4 (129.8, 402.7)	-7.0 (-126.5, 98.4)	316.8 (200.7, 498.8)	-75.0 (-119.8, 90.1)	0.70	0.83	0.12	0.40
HOMA-IR[7]	5.5 (3.4, 8.5)	-0.3 (-2.3, 1.5)	4.3 (2.9, 9.2)	-0.7 (-2.3, 0.6)	0.68	0.60	0.95	0.49
Matsuda ISI[8]	1.1 (0.7, 1.6)	0.1 ± 0.8	1.1 (0.6, 2.0)	0.1 ± 0.6	0.95	0.99	0.19	0.98
AUC Insulin (μU/mL x min)[9]	20,885 (13,812, 32,053)	-2,135 (-7,292, 5,244)	23,047 (15,727, 38,691)	-1,061 (-6,896, 4,801)	0.72	0.80	0.37	0.51
Insulinogenic Index[7]	5.7 (4.1, 8.5)	0.6 (-1.3, 2.4)	5.6 (4.6, 8.2)	0.3 (-1.5, 1.0)	0.56	0.56	0.91	0.44
Oral DI[10]	6.4 (4.3, 8.7)	1.5 ± 5.2	6.8 (3.0, 11.2)	0.6 ± 4.6	0.47	0.41	0.21	<0.001

Values are means ± standard deviations or medians (25th, 75th percentiles) for non-normally distributed variables

Raw: adjusted for baseline value of the outcome of interest

Adjusted: raw model adjusted for sex, change in body mass index, and change in physical activity

GEE: generalized estimating equation analysis of adjusted model further adjusting for sibling clusters

1 Sample size: Δ total sugar (%E) <0 (n=60), Δ total sugar (%E) ≥ 0 (n=26), sibling cluster (n=10)

2 Sample size: Δ total sugar (%E) <0 (n=60), Δ total sugar (%E) ≥ 0 (n=24), sibling cluster (n=10)

3 Sample size: Δ total sugar (%E) <0 (n=60), Δ total sugar (%E) ≥ 0 (n=25), sibling cluster (n=10)

4 Sample size: Δ total sugar (%E) <0 (n=57), Δ total sugar (%E) ≥ 0 (n=24), sibling cluster (n=9)

5 Sample size: Δ total sugar (%E) <0 (n=55), Δ total sugar (%E) ≥ 0 (n=23), sibling cluster (n=8)

6 Sample Size: Δ total sugar (%E) <0 (n=59), Δ total sugar (%E) ≥ 0 (n=27), sibling cluster (n=9)

7 Sample Size: Δ total sugar (%E) <0 (n=58), Δ total sugar (%E) ≥ 0 (n=25), sibling cluster (n=9)

8 Sample Size: Δ total sugar (%E) <0 (n=56), Δ total sugar (%E) ≥ 0 (n=25), sibling cluster (n=8)

9 Sample size: Δ total sugar (%E) <0 (n=53), Δ total sugar (%E) ≥ 0 (n=24), sibling cluster (n=8)

10 Sample size: Δ total sugar (%E) <0 (n=55), Δ total sugar (%E) ≥ 0 (n=24), sibling cluster (n=8)

Abbreviations: ANCOVA: analysis of covariance, AUC: area under the curve, GEE: generalized estimating equation, HbA1c: glycated hemoglobin, HOMA-IR: homeostatic model assessment of insulin resistance, ISI: insulin sensitivity index, oral DI: oral disposition index

Table S3. The effect of total sugar intake on serum lipids and blood pressure in the primary analysis.

	**Δ total sugar (%E) <0**		**Δ total sugar (%E) ≥ 0**		**ANCOVA**			**GEE**
	Baseline	Change	Baseline	Change	Raw	Adjusted	Sex interaction	Adjusted
Cholesterol (mg/dL)[1]	143.0 (127.8, 159.0)	-1.8 ± 13.7	147.5 (135.8, 153.0)	0.9 ± 13.7	0.28	0.42	0.42	0.58
Triglycerides (mg/dL)[2]	94.0 (78.0, 128.0)	0.0 (-14.5, 19.0)	107.0 (83.5, 144.5)	7.0 (-11.0, 35.0)	0.047	0.049	0.91	0.06
LDL cholesterol (mg/dL)[1]	81.6 ± 24.0	-2.2 ± 12.7	80.4 ± 19.0	-1.1 ± 9.3	0.74	1.0	0.88	0.70
HDL cholesterol (mg/dL)[3]	39.0 (35.0, 44.0)	0.3 ± 4.6	40.0 (36.0, 44.5)	-1.4 ± 3.9	0.20	0.27	0.11	0.19
Cholesterol:HDL[4]	3.7 ± 1.0	-0.1 ± 0.5	3.6 ± 1.0	0.1 ± 0.4	0.046	0.08	0.45	0.08
Systolic BP (mmHg)[5]	116.0 ± 12.2	-2.5 ± 9.4	115.4 ± 8.7	-2.0 ± 8.7	0.87	0.92	0.97	0.94
Diastolic BP (mmHg)[6]	68.8 ± 6.8	-1.0 (-5.1, 3.5)	69.4 ± 6.1	-0.5 (-2.4, 2.4)	0.43	0.42	0.77	0.38

Values are means ± standard deviations or medians (25th, 75th percentiles) for non-normally distributed variables

Raw: adjusted for baseline value of the outcome of interest

Adjusted: raw model adjusted for sex, change in body mass index, and change in physical activity

*GEE: generalized estimating equation analysis of adjusted model further adjusting for sibling clusters (n=10)

1 Sample Size: Δ total sugar (%E) <0 (n=60), Δ total sugar (%E) ≥ 0 (n=24), sibling cluster (n=10)

2 Sample Size: Δ total sugar (%E) <0 (n=59), Δ total sugar (%E) ≥ 0 (n=23), sibling cluster (n=10)

3 Sample Size: Δ total sugar (%E) <0 (n=60), Δ total sugar (%E) ≥ 0 (n=22), sibling cluster (n=10)

4 Sample size: Δ total sugar (%E) <0 (n=61), Δ total sugar (%E) ≥ 0 (n=24), sibling cluster (n=10)

5 Sample size: Δ total sugar (%E) <0 (n=62), Δ total sugar (%E) ≥ 0 (n=26), sibling cluster (n=10)

6 Sample size: Δ total sugar (%E) <0 (n=60), Δ total sugar (%E) ≥ 0 (n=26), sibling cluster (n=10)

Abbreviations: ANCOVA: analysis of covariance, BP: blood pressure, GEE: generalized estimating equation, HDL: high-density lipoprotein, LDL: low-density lipoprotein

Table S4. The effect of total sugar intake on adipokines and inflammatory markers in the primary analysis.

	Δ total sugar (%E) <0		Δ total sugar (%E) ≥ 0		ANCOVA			GEE
	Baseline	Change	Baseline	Change	Raw	Adjusted	Sex interaction	Adjusted
CRP[1]	2.3 (0.8, 6.2)	-0.1 (-1.0, 1.1)	1.3 (0.5, 4.0)	-0.1 (-0.5, 0.7)	0.67	0.77	0.62	0.66
IL-6[2]	8.1 (5.1, 31.5)	-0.6 (-2.7, 4.4)	9.0 (5.7, 59.4)	2.0 (-3.2, 5.9)	0.21	0.25	0.17	0.37
TNF-alpha[3]	3.0 ± 1.0	0.1 (-0.3, 0.9)	2.7 ± 1.1	0.2 (-0.2, 0 .4)	0.06	0.02	0.43	0.02
MCP-1[4]	105 ± 29	2 ± 22	95 ± 30	6 ± 24	0.72	0.78	0.39	0.84
Leptin[5]	7520 (4406, 10699)	-407 ± 2640	6176 (3524, 10492)	124 ± 2531	0.56	0.83	0.84	0.80

Values are means ± standard deviations or medians (25th, 75th percentiles) for non-normally distributed variables

Raw: adjusted for baseline value of the outcome of interest

Adjusted: raw model adjusted for sex, change in body mass index, and change in physical activity

GEE: generalized estimating equation analysis of adjusted model further adjusting for sibling clusters

[1] Sample size: Δ total sugar (%E) <0 (n=55), Δ total sugar (%E) ≥ 0 (n=24), sibling cluster (n=8)

[2] Sample size: Δ total sugar (%E) <0 (n=36), Δ total sugar (%E) ≥ 0 (n=18), sibling cluster (n=6)

[3] Sample size: Δ total sugar (%E) <0 (n=58), Δ total sugar (%E) ≥ 0 (n=26), sibling cluster (n=9)

[4] Sample size: Δ total sugar (%E) <0 (n=60), Δ total sugar (%E) ≥ 0 (n=26), sibling cluster (n=9)

[5] Sample size: Δ total sugar (%E) <0 (n=60), Δ total sugar (%E) ≥ 0 (n=27), sibling cluster (n=9)

Abbreviations: ANCOVA: analysis of covariance, BMI: body mass index, CRP: C-reactive protein, GEE: generalized estimating equations,

IL-6: interleukin-6, MCP-1: monocyte chemoattractant protein-1

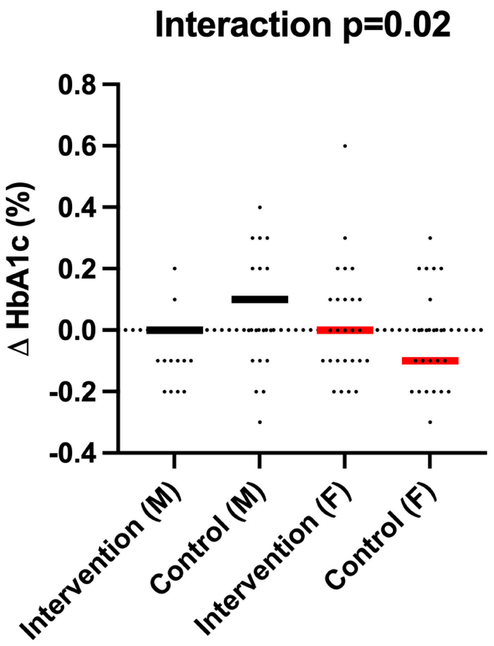

Figure S1. Changes in HbA1c differ by participant sex. Changes in HbA1c (post-intervention minus the value at baseline) for participants by intervention group and participant sex. Each participant's change variable is represented by a solid dot. The medians are represented by horizontal bars. The p-value for sex-by-treatment interaction in the analysis of covariance adjusted for the baseline HbA1c, change in body mass index, and change in physical activity is displayed at the top of the plot. ANCOVA: analysis of co-variance, HbA1c: glycated hemoglobin,

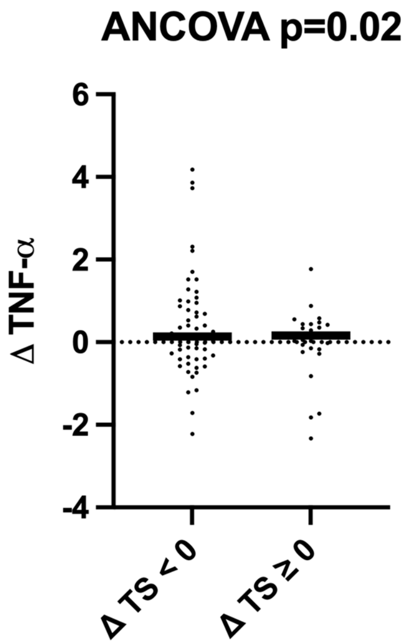

Figure S2. Changes (post-intervention minus the value at baseline) in TNF-α for participants with total sugar reduction as a percent of energy ($\Delta\, TS < 0$) and those without ($\Delta\, TS \geq 0$). Each participant's change variable is represented by a solid dot. The medians are represented by horizontal bars. The p-value for the analysis of covariance adjusted for the change in body mass index and change in physical activity is displayed at the top of the plot. ANCOVA: analysis of co-variance**,** TNF-α:Tumor Necrosis Factor –Alpha, TS: Total Sugar
